# Plasmid-like dynamics of persistent RNA viruses in the host fungal population

**DOI:** 10.1128/jvi.00582-25

**Published:** 2025-07-31

**Authors:** Yuto Chiba, Seiyo Watanabe, Ayano Ikeda, Shuhei Miyashita, Daisuke Hagiwara, Syun-ichi Urayama

**Affiliations:** 1 Laboratory of Fungal Interaction and Molecular Biology (donated by IFO), Department of Life and Environmental Sciences, University of Tsukuba 13121, Tsukuba, Ibaraki, Japan; 2 School of Agriculture, Meiji University 98289 , Kawasaki, Kanagawa, Japan; 3 Graduate School of Agricultural Science, Tohoku University 13101, Sendai, Miyagi, Japan; 4 Microbiology Research Center for Sustainability (MiCS), University of Tsukuba 13121, Tsukuba, Ibaraki, Japan; 5 Medical Mycology Research Center, Chiba University, Chuo-ku, Chiba, Japan; 6 Tsukuba Institute for Advanced Research (TIAR), University of Tsukuba, Tsukuba, Ibaraki, Japan; Emory University School of Medicine, Atlanta, Georgia, USA

**Keywords:** fungal RNA virus, mycovirus, population dynamics, plasmid

## Abstract

Unlike the well-known acute or chronic animal and plant RNA viruses, most fungal RNA viruses (RNA mycoviruses) have a persistent life cycle. They lack an extracellular infection route and coexist with their hosts for a lifetime. RNA mycoviruses affect various biological properties of host fungi and are widespread across all major fungal groups. The frequency of fungal isolates harboring RNA mycoviruses ranges from a few percent to several tens of percent, indicating their high persistence within ecosystems. However, the dynamics of RNA mycoviruses and the maintenance mechanisms within host populations remain largely unknown. Here, we developed an experimental laboratory model system to examine the dynamics of mycoviruses within clonal host populations and to clarify the mechanisms. In our experimental system, we used two mycoviruses infecting *Aspergillus fumigatus*. Using this system, we discovered that viral prevalence changed according to environmental conditions (temperature or fungicide exposure). The driving forces behind these prevalence changes were viral horizontal transmission between isogenic strains through hyphal fusion, viral loss, and viral impact on host growth. Our findings indicated that the dynamics of mycoviruses are similar to those of plasmids in bacterial populations. This analogy implied an uncovered role of mycoviruses as a switching factor for fungal adaptation to changing environments.

**IMPORTANCE:**

While RNA viruses are generally known for their ability to infect cells from the extracellular environment, a substantial diversity of RNA viruses, particularly those that persistently infect fungi, lack such infectivity. The ecological success and evolutionary maintenance of these persistent RNA viruses remain poorly understood. In this study, we experimentally demonstrate how a non-infectious RNA virus is transmitted and stably maintained within specific fungal host lineages. The revealed mechanism resembles the inheritance strategies of plasmids, highlighting a fundamentally different viral lifestyle that does not rely on extracellular horizontal transmission. These findings advance our understanding of virus-host interactions beyond the classical infection model and shed light on the evolutionary flexibility of RNA viruses.

## INTRODUCTION

**Fig 1:**
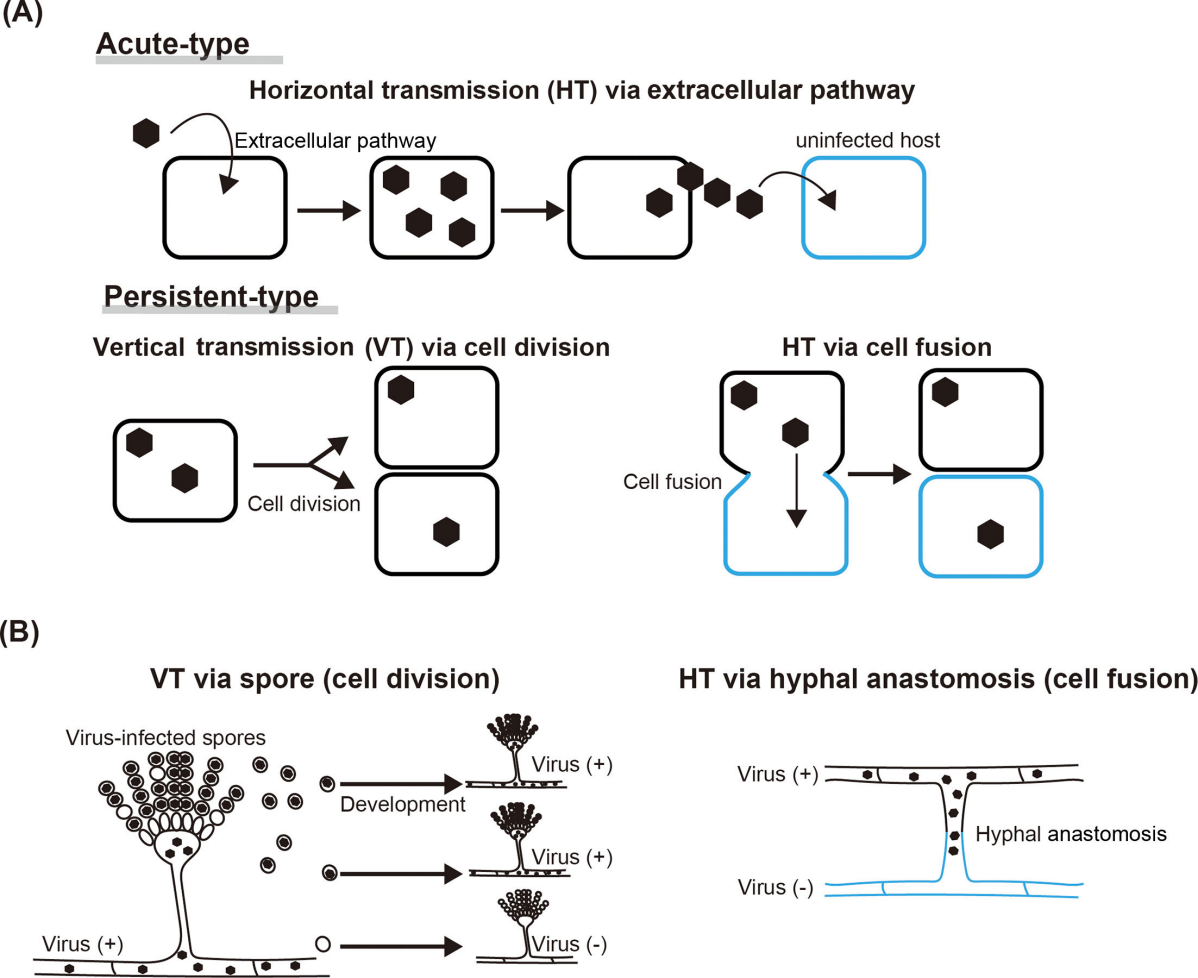
Transmission routes of acute-type and persistent-type RNA viruses. (**A**) Major transmission routes of acute-type and persistent-type RNA viruses. (**B**) Primary modes of mycovirus transmission.

Many animal, plant, and bacterial RNA viruses share a common feature: they are mainly transmitted horizontally via extracellular transmission (1–5). RNA viruses with this type of life cycle are called acute-type RNA viruses (Fig. 1A) (6–9). In contrast, fungal RNA viruses (known as mycoviruses) and several plant RNA viruses are thought to lack an extracellular infection route and persistently infect their hosts for a lifetime. These RNA viruses are called persistent-type RNA viruses (Fig. 1A) (6–9). It is known that mycoviruses are mainly transmitted vertically through cell division in addition to cell fusion, called hyphal anastomosis (Fig. 1B) (10–13). The ratio of virus-infected isolates within a single fungal species is 7% to 80% (11, 14). These findings indicate that mycoviruses are widespread in the fungal kingdom. Notably, in nature, a single mycovirus infects a single host species, except for a few cases (15–17).

The effects of mycoviruses on their host fungi have been extensively studied. Several mycoviruses are known to influence the biological properties of their host fungus, such as growth, metabolic processes, and environmental stress tolerance (18–22). Additionally, mycoviruses indirectly modify the biological interactions between hosts and other organisms. For example, some mycoviruses reduce or increase the virulence of their host phytopathogenic fungi (10, 19, 23, 24). Interestingly, a mycovirus can convert the fungal host from a plant-pathogenic fungus to a non-pathogenic endophyte (22).

The survival of obligate parasitic elements like viruses is critically dependent on the survival of their hosts and maintenance within the host population. Extensive research has been conducted on how acute-type RNA viruses, such as severe acute respiratory syndrome coronavirus 2, are maintained within host populations, and numerous sophisticated models have been proposed (25–28). In these models, horizontal transmission is the main factor. In contrast, there are fewer studies on the survival of persistent-type RNA viruses (29), whereas plasmids, mobile genetic elements commonly found in bacteria, have a life cycle similar to that of persistent-type RNA viruses (30–32). In plasmids, it was reported that the primary factors driving their dynamics are horizontal transmission, vertical transmission via cell division, loss, and impact on host fitness (30). Therefore, we hypothesized that factors such as horizontal transmission through mycelial fusion, vertical transmission via cell division, loss during cell division, and changes in the fitness of host fungi are potential contributors to mycovirus maintenance.

To investigate the contribution of these factors, we focused on a widely distributed and well-characterized fungal species, *Aspergillus fumigatus*. RNA mycoviruses infecting *A. fumigatus* have also been extensively studied (14, 33). We selected two phylogenetically distinct RNA viruses: Aspergillus fumigatus RNA virus 1 (AfuRV1) and Aspergillus fumigatus narnavirus 2 (AfuNV2), which belong to the viral phyla *Kitrinoviricota* and *Lenarviricota*, respectively (33, 34). AfuRV1 is an encapsidated positive-sense single-stranded RNA (+ssRNA) virus with a deca-segmented genome (34). In contrast, AfuNV2 is a + ssRNA virus with a tri-segmented genome, and no capsid protein has been identified to date (33). We chose these viruses because *A. fumigatus* strains harboring AfuRV1 or AfuNV2 exhibited stable and distinguishable phenotypes compared to their virus-cured counterparts, making them suitable models for investigating viral maintenance within clonal host populations.

Here, we present a framework for observing the maintenance of persistent-type RNA viruses using a simplified laboratory experiment. We mixed spores obtained from *A. fumigatus* with or without mycovirus and then monitored the percentage change in mycovirus-infected spores under different conditions. At the same time, we also monitored the transmission and loss of mycovirus using genetic markers.

## MATERIALS AND METHODS

### Fungi and viruses used in this study

In this study, we used two *A. fumigatus* strains infected with RNA viruses: IFM 63431 (infected with AfuNV2) and IFM 63439 (infected with AfuRV1) (33). Their derivatives were also used. First, virus-cured IFM 63431 and IFM 63439 were obtained using antiviral drugs (35). Virus-infected strains were cultured on a potato dextrose agar (PDA) plate with antiviral ribavirin and 2′-C-methyladenosine, respectively. After that, we collected spores and tested the virus infection in each colony derived from the collected spores, as described below. Colonies with no detection of viruses were isolated and used as virus-free strains in this study. Second, we transformed the virus-cured strains using pCB1004, as described previously (36), to add a hygromycin resistance marker (Hyg^R^) into the chromosome. In brief, pCB1004 carrying the Hyg^R^ cassette was introduced into protoplasts of virus-cured IFM 63431 and IFM 63439 using PEG6000 (FUJIFILM Corporation, Tokyo, Japan). The candidates of Hyg^R^ transformants were screened by minimal medium (37) supplemented with hygromycin B (200 µg/mL), and then the candidates were isolated on PDA slant. The Hyg^R^ transformants were obtained after confirmation by PCR.

### Preparation of the initial population (spore mixture)

We prepared the initial spore suspension (G0 population). Virus-infected and -free strains were cultured on PDA slants separately at 37°C for 2–5 days. After that, spores of each strain were collected with 0.05% Tween 20 solution (Nacalai Tesque, Kyoto, Japan), and the collected spores (1 × 10^5^) were suspended in 10 mL of PDA before the medium solidified, independently. The PDA with spores was poured into a sterilized and dried 100 mL flask. They were cultured at 37°C for 2 days, and 0.05% Tween 20 solution was added to the flask and suspended to harvest spores from the entire surface of the colonies. The collected spore suspension was filtered using a Miracloth to remove fungal mycelia. Then, the initial spore suspension was generated by mixing the spore suspensions (5 × 10^7^ spores/mL) of the virus-infected and -free strains. Virus prevalence in the initial population was adjusted by changing the mixing ratio of virus-infected and -free spore suspensions. The vertical transmission rates of AfuRV1 and AfuNV2 are approximately 96% and 99%, respectively, at 37°C (Table S1).

### Serial passage of the AfuRV1-infected population

**Fig 2:**
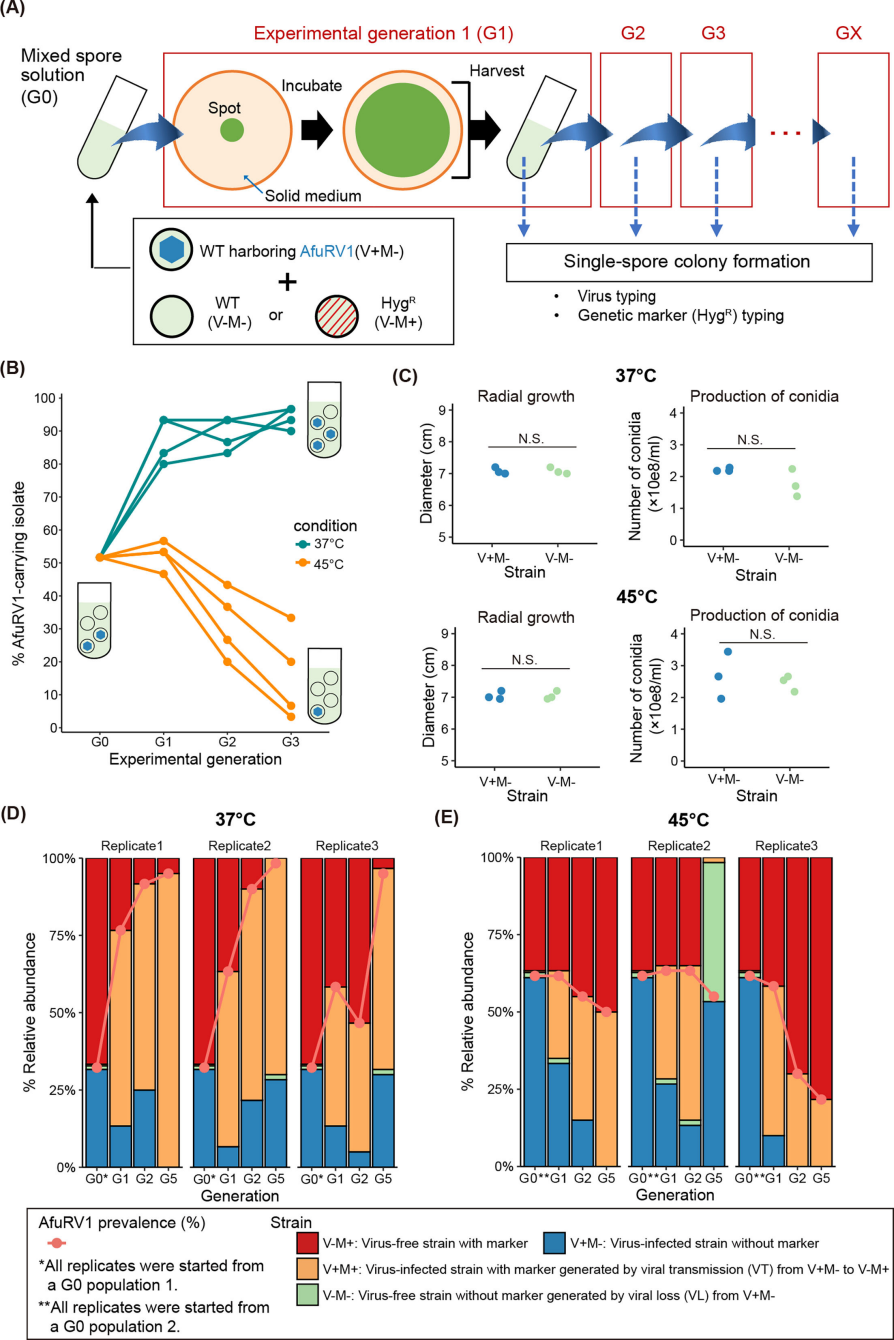
Temperature-dependent dynamics of AfuRV1 prevalence and its responsible factors. (**A**) Experimental procedure for serial passage experiments of AfuRV1-infected populations at 37°C or 45°C. Treatments are shown for a single replicate population. (**B**) Dynamics of AfuRV1 prevalence under two different temperatures. Lines connect each of the four replicate populations per treatment across time. Four populations were passaged independently from an initial spore suspension. We tested 120 single colonies (30 colonies/replicate population). *P*-values were calculated using Fisher’s exact test and adjusted for multiple comparisons by Bonferroni’s correction. (**C**) Growth assay of AfuRV1-infected and -cured strains. Three independent cultures were examined. Dots indicate each biological replicate. Welch’s *t*-test was used to compare radial growth and conidia production between AfuRV1-infected and -cured strains (*P < 0.01; N.S. *P* > 0.05). Monitoring of AfuRV1 horizontal transmission and loss at (**D**) 37°C or (**E**) 45°C. The stacked bar graph represents the relative abundance of each strain. One day after the colonies reached the edge of the flask (37°C: day 4, 45°C: day 6), we harvested spores and initiated the next experimental generation. We tested 180 single colonies (60 colonies/replicate population). *P*-values were calculated using Fisher’s exact test.

The initial population (G0 population) was cultured for more than three experimental generations at 37°C or 45°C (Fig. 2A). In detail, 2 µL of the initial spore suspension (1 × 10^5^ spores) was dropped onto the center of PDA on the bottom of a 100 or 200 mL flask and cultured at 37°C or 45°C. One day after the colony reached the edge of the flask (37°C: day 6, 45°C: day 8), spores were harvested from the entire surface of the colony (G1 population). The 2 µL of the G1 spore suspension (1 × 10^5^ spores) was inoculated onto fresh PDA on the bottom of a 100 or 200 mL flask to initiate the next experimental generation (G2). AfuRV1 and Hyg^R^ prevalence in each generation was observed as described below.

### Single passage of the AfuNV2-infected population

The initial population was inoculated at the center of the PDA plate containing 10 µg/mL nystatin or 0.1 µg/mL voriconazole dissolved in dimethylsulfoxide (DMSO). As a control experiment, the initial population was also inoculated on a PDA plate containing DMSO alone without fungicides. After incubation at 37°C for 14 days, we collected mycelial plugs from spore-producing areas in the colonies and harvested conidia from the plugs with 0.05% Tween 20. AfuNV2 and Hyg^R^ prevalence in each sample was observed as described below. Three populations were cultured independently from an initial spore suspension, and 90 single colonies were tested (30 colonies/replicate population).

### Detection of viral infection in each single colony

To observe virus prevalence, we checked the presence or absence of the virus in each single colony. Firstly, we spread 200 µL of the spore suspensions (including about 50 spores) on PDA or yeast extract peptone dextrose agar (YPDA: Bacto Yeast Extract 1%, Bacto Peptone [BD] 2%, D-(+)- glucose [Nacalai Tesque] 2%, agar 1.5%) media to obtain single colonies. After incubation for 20 h at 37°C, we checked the virus infection using direct one-step reverse transcription-PCR (RT-PCR) analysis as described previously (38). One-step RT-PCR was conducted using a PrimeScript One Step RT-PCR Kit Ver.2 (Takara, Japan) or Super-Script III One-Step RT-PCR System with Platinum Taq (Invitrogen) and virus-specific primer pairs (Table S2). Reagent compositions and PCR cycles followed the manufacturer’s protocol. The PCR products were subjected to 1% agarose gel electrophoresis and visualized with GelRed staining (Biotium, Fremont, CA, USA).

### Detection of the Hyg^R^ marker gene in each single colony

To judge if the colony was derived from the recipient strain, we detected the Hyg^R^ gene. For the AfuRV1 experiment, we detected the Hyg^R^ gene by PCR analysis. In the detection of PCR products, we used a real-time PCR instrument to expedite detection. The relative fluorescence units at the PCR reaction endpoint determined the presence or absence of the marker gene. Real-time PCR reaction was performed using THUNDERBIRD Next SYBR qPCR Mix (TOYOBO, Osaka, Japan) and Hyg^R^ gene-specific primer pairs (Table S2). Reagent compositions and PCR cycles followed the manufacturer’s protocol. For the AfuNV2 experiment, we determined the presence or absence of the marker gene by Hyg^R^ phenotype. The colonies tested for virus infection were transferred to PDA media containing hygromycin B (200 µg/mL). After incubation at 37°C for 1 or 2 days, we checked mycelia growth on the selection media.

### Fitness assays

We compared the colony growth and number of conidia between AfuRV1-infected and -free strains at 37°C and 45°C. Colony growth was evaluated by quantifying colony diameter. Conidia (1 × 10^5^) were inoculated on the center of a PDA plate and incubated at 37°C for 3 days or 45°C for 4 days, and the colony diameter was measured. To compare sporulation, 1 × 10^5^ conidia were mixed with 8 mL PDA before the medium solidified in a 100 mL flask and incubated at 37°C for 4 days or 45°C for 6 days. Then, the produced conidia were harvested with 15 mL of 0.05% Tween 20 solution, and the number of conidia was counted with a hemocytometer (Watson, Maidstone, UK).

For colony growth tests of AfuNV2-infected and -free strains, 1 × 10^5^ conidia were inoculated on the center of a PDA plate with the following concentrations of antifungal compounds: 0.1 µg/mL voriconazole and 10 µg/mL nystatin. These antifungal agents target ergosterol, the fungal membrane sterol, but the mechanisms of action are different (39). Voriconazole is a triazole antifungal agent that inhibits the biosynthesis of ergosterol. Nystatin is a polyene antifungal agent that causes leakage of cellular contents by binding to the ergosterol itself instead of inhibiting ergosterol biosynthesis. These antifungal agents were dissolved in DMSO and added to the PDA, respectively. DMSO without antifungal compounds was used for the control PDA plate. Colony diameter was measured after incubation at 37°C for 3 days. For nystatin, the culture time was 5 days. Moreover, we compared the sporulation ability between AfuNV2-infected and free strains under the abovementioned conditions. The number of conidia was counted in the same procedure as AfuRV1. The culture time in each condition was the same for colony growth tests.

### Statistical analysis

All statistical analyses were conducted in R version 4.3.1. Fisher’s exact test was used to assess the differences in virus prevalence between before and after culture. In this analysis, all replications were combined into a single data set for each generation, and Fisher’s exact test was performed using the RVAideMemoire package in R software (40). Welch’s *t*-test was used to examine the differences in growth between virus-infected and -cured strains. Statistical significance was defined as *P* < 0.05.

## RESULTS

### Temperature-dependent dynamics of AfuRV1 in the clonal host population

To investigate the dynamics of persistent RNA viruses, we co-cultured AfuRV1-infected and -cured strains of *A. fumigatus* under two temperatures and examined changes in RNA virus prevalence (Fig. 2A). The population used in this experiment is clonal since AfuRV1-infected and -cured strains were isogenic lines (see Materials and Methods). An initial population (G0 population) with around 50% prevalence of AfuRV1 was cultured for three experimental generations at 37°C or 45°C, the growth temperatures for this fungus in the natural environment (41). The AfuRV1 prevalence was observed in each temperature and experimental generation. The AfuRV1 prevalence increased significantly at 37°C (Fisher’s exact test, G0 vs G3, *P* < 0.01) (Fig. 2B, green line). In contrast, the AfuRV1 prevalence decreased significantly at 45°C (Fisher’s exact test, G0 VS G3, *P* < 0.01) (Fig. 2B, orange line). These results suggest that AfuRV1 shows temperature-dependent prevalence dynamics within a host population.

### Mechanisms responsible for the AfuRV1 prevalence dynamics

Since no significant differences in growth rates and conidia formations were observed between the virus-infected and cured strains at each temperature (Fig. 2C), we focused on horizontal transmission and the loss of AfuRV1 as the driving force behind the prevalence change. To monitor horizontal transmission and loss of AfuRV1, we co-cultured an AfuRV1-infected strain without the Hyg^R^ gene (V+M−) and an AfuRV1-cured strain with the Hyg^R^ gene (V−M+) (Fig. 2A). When horizontal transmission of AfuRV1 occurs by hyphal anastomosis, a new strain harboring both AfuRV1 and the Hyg^R^ gene (V+M+ ) emerges. In contrast, when the AfuRV1-infected strain (V+M−) loses AfuRV1, another new strain (V−M−) arises.

An initial population with around 50% prevalence of AfuRV1 was passaged five times at 37°C in a 100 mL flask with solid media. As a result, a strain with both AfuRV1 and the Hyg^R^ gene (V+M+ ) emerged and significantly increased (Fisher’s exact test, G0 VS G5, *P* < 0.01) (Fig. 2D, orange). Along with the increase of this strain, total AfuRV1 prevalence significantly increased (Fisher’s exact test, G0 VS G5, *P* < 0.01) (Fig. 2D, orange line) as observed in the markerless experiments (Fig. 2B, green line). These results indicate that AfuRV1 was transmitted horizontally in this condition, and this transmission was the primary cause of increased AfuRV1 prevalence.

**Fig 3:**
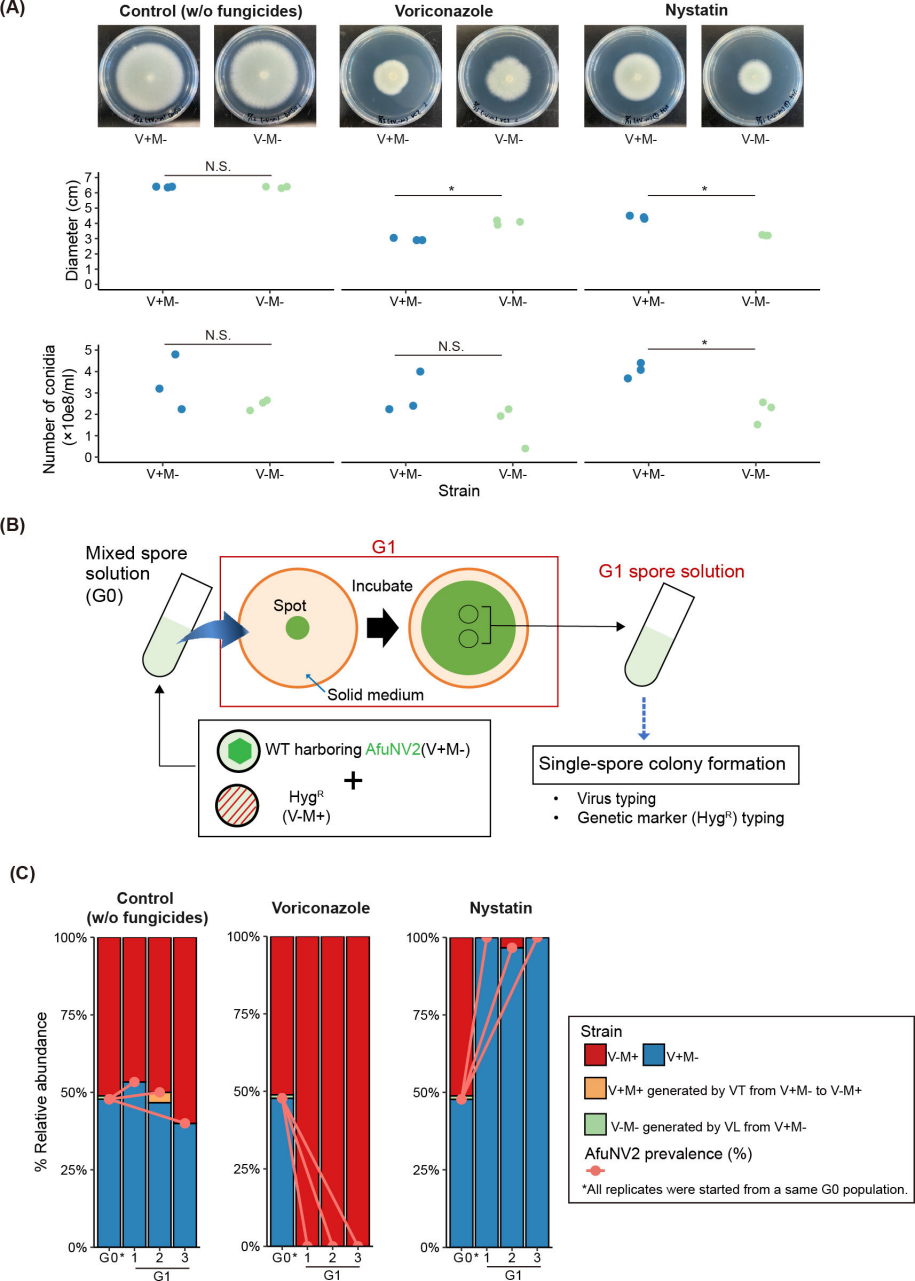
Dynamics of AfuNV2 prevalence under antifungal agents. (**A**) Growth assay and colony morphology of AfuNV2-infected and -cured strains on PDA plates with nystatin and voriconazole. Three independent cultures were examined. Dots indicate each biological replicate. Welch’s *t*-test was used to compare radial growth between AfuNV2-infected and -free strains (*P < 0.05; N.S. *P* > 0.05). (**B**) Experimental procedure for observation of AfuNV2 prevalence. Treatments are shown for a single replicate population. (**C**) AfuNV2 prevalence and relative abundance of each strain. In the comparison of AfuNV2 prevalence between before and after culture, *P*-values were calculated using Fisher’s exact test and adjusted for multiple comparisons by Bonferroni’s correction.

On the other hand, at 45°C, AfuRV1 prevalence tended to decrease (Fig. 2E, orange lines). In replicate 2, the V−M− strain increased (Fig. 2E, green), indicating that the loss of AfuRV1 occurred during this experiment. Supporting this interpretation, we observed sectoring phenomena associated with the emergence of the V−M− strain from the V+M− strain on plate media at 45°C but not at 37°C (Fig. S1). Since the vertical transmission rate of AfuRV1 to spores is not 100% (Table S1), the observed sectoring may have resulted from a few virus-free cells that were already present before incubation at 45°C, rather than from the loss of AfuRV1 during incubation. To address this possibility, we examined virus-infected colonies derived from single-spore isolation (Fig. S2). As a result, sectoring was still observed at 45°C even in colonies derived from single spores. Among the 14 colonies tested, sectoring occurred in 4. These results indicate that loss of AfuRV1 is one of the factors responsible for the decreased AfuRV1 prevalence at 45°C. When we examined AfuRV1 prevalence in spores obtained from non-sectoring regions incubated at 45°C and 37°C, no significant difference was found between the two temperatures (Fisher’s exact test, total at 45°C vs total at 37°C, *P* > 0.05) (Table S1). This result suggests that the significant loss of AfuRV1 primarily occurs in the sectoring regions. In replicates 1 and 3, the V−M+ strain increased (Fig. 2E, red) instead of the V−M− strain (Fig. 2E, green). These data suggest the presence of other factor(s). It is presumed that there are subtle phenotypic differences between V+ and V− strains. Although there were no significant differences in growth rates and spore formation abilities between V+ and V− strains (Fig. 2C and Fig. 3), the virus-free areas corresponding to the sectoring area seemed to grow faster than the infected areas in a virus-infected colony (Fig. S1). At the same time, horizontal transmission also occurred at 45°C, as the V+M+ strain increased after cultivation (Fig. 2E, orange). Together, our observations suggest that at least three factors contribute to the prevalence dynamics at 45°C; that is, loss of virus, slightly higher fitness of V− strains, and horizontal transmission, implying that the degree of contribution of each factor varies between replicates.

In summary, horizontal transmission plays a significant role in driving prevalence dynamics. Loss of viruses and subtle phenotypic differences were also contributing factors.

### Dynamics of AfuNV2 prevalence under antifungal agents

Since the fitness difference observed in the AfuRV1 experiment was subtle, the AfuRV1 experiments did not provide clear evidence that viral effects on host fitness contribute to the prevalence dynamics. Hence, we used AfuNV2-infected and -cured strains of *A. fumigatus*. They showed similar growth on plate media without antifungal agents (Fig. 3A). However, in the presence of voriconazole, the AfuNV2-cured strain exhibited faster growth than the AfuNV2-infected strain; whereas, in the presence of nystatin, the AfuNV2-infected strain exhibits faster growth and higher conidia production than the AfuNV2-cured strain (Fig. 3A). To examine the impact of fitness on AfuNV2 prevalence, we observed AfuNV2 prevalence under the pressure of antifungal agents (Fig. 3B and C). In this experiment, we also used an AfuNV2-infected strain without the Hyg^R^ gene (V+M−) and an AfuNV2-cured strain with the Hyg^R^ gene (V−M+) to monitor the horizontal transmission and loss of AfuNV2.

In the control condition, we did not observe any significant changes in either AfuNV2 prevalence or relative abundance of each strain (Fig. 3C, left). Meanwhile, these indicators fluctuated under the influence of antifungal agents (Fig. 3C). In the presence of voriconazole, AfuNV2 prevalence significantly decreased (Fisher’s exact test, G0 VS G1, *P* < 0.01) (Fig. 3C, center) due to a fitness disadvantage for the host. Conversely, in the presence of nystatin, AfuNV2 prevalence significantly increased (Fisher’s exact test, G0 VS G1, *P* < 0.01) (Fig. 3C, right) due to a fitness advantage for the host. In these experiments, the strains that emerged from viral transmission or loss were rarely detected. In summary, viral effects on host fitness play a crucial role in driving prevalence dynamics.

## DISCUSSION

Previous hypotheses have attempted to explain the long existence of persistent RNA viruses in plants, parasitic protozoa, and fungi (29, 42); however, these explanations have lacked experimental evidence on their prevalence dynamics and mechanisms. Here, we discovered that mycoviruses exhibit dynamic prevalence within a clonal fungal population in response to environmental changes, such as temperature fluctuations and fungicide exposure. Furthermore, we experimentally demonstrated that horizontal transmission, viral loss, and viral impact on host fitness contribute to these prevalence dynamics. These findings represent the first theoretical framework explaining the persistence of RNA viruses and mark a significant milestone in our understanding of persistent RNA viruses.

**Fig 4:**
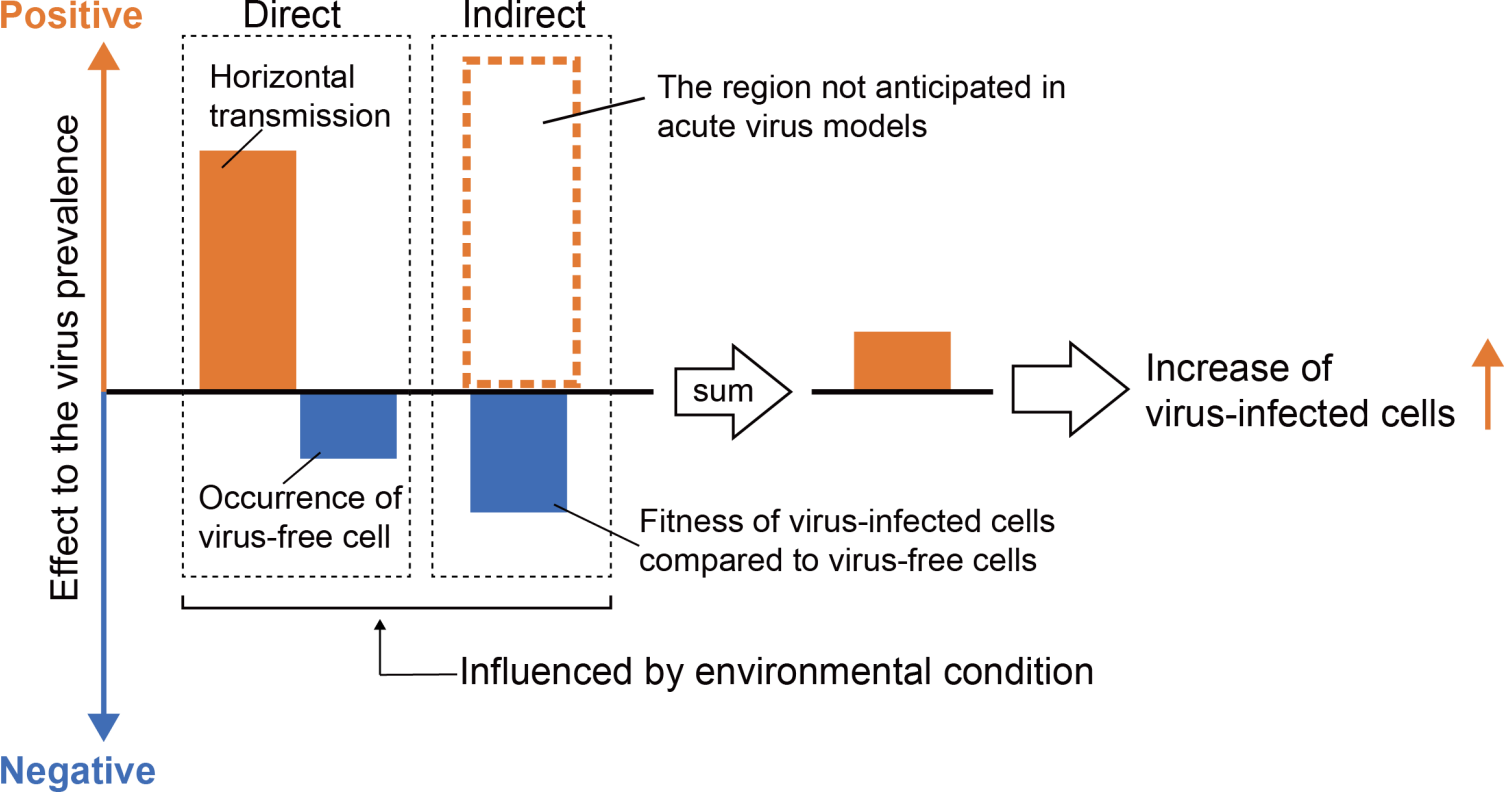
A proposed explanation for the process of mycovirus prevalence fluctuation in a clonal host population.

Based on our results, we suggest a framework to explain the fluctuation of mycovirus prevalence in the clonal host population (Fig. 4). The factors contributing to mycovirus prevalence dynamics are independent of each other and can be divided into two types. Horizontal transmission and loss of viruses directly impact virus prevalence. Meanwhile, viral effects on host fitness indirectly contribute to the prevalence dynamics through competition between virus-infected and -free strains. The sum of these factors determines the increase or decrease of mycovirus prevalence. For example, in the case of AfuRV1 prevalence at 45°C, the intensity of viral effects on host fitness was almost zero (Fig. 2C). Viral loss was a stronger factor than horizontal transmission (Fig. 2E and Fig. 1). The total value of these factors was negative, and thus, AfuRV1 prevalence decreased. The viral loss under high-temperature conditions is also reported in other mycoviruses (43–45). In general, when vertical transmission is the dominant mode of transmission, the survival of parasites is tightly linked with their host’s survival. Theoretically, it was predicted that these parasites favor low virulence and mutual benefit for their host fitness (46–48). Actually, many positive effects have been reported for mycoviruses (19, 24, 49–51). However, adverse effects, such as hypovirulence and growth reduction, are sometimes observed for mycoviruses (10, 13). These observations conflict with the dominant transmission mode of mycoviruses (12). In our framework, the long existence of persistent RNA viruses with adverse effects can be explained by factors that increase virus prevalence. When their adverse effects on host fitness (a factor that decreases virus prevalence) are balanced by the opposite factors, these viruses can be maintained in their host population. Thus, these viruses may have higher horizontal transmission rates than previously thought and/or condition-dependent positive effects on host fitness. This interpretation is consistent with previous theoretical and experimental studies (17, 52–55).

This framework can also be considered an extension of the acute-type RNA virus model. This has evolved from a model that considers only negative impacts on fitness (pathogenicity) to one that includes potential positive impacts (Fig. 4). This simple extension allows for discussing maintenance mechanisms for all RNA viruses, including the persistent type, on the same foundation, not just those of pathogenic RNA viruses.

Interestingly, the prevalence of the virus did not reach either 100% or 0% in experiments that utilized AfuRV1 (Fig. 2B; Fig. S4). The reason might be that the efficiency of horizontal transmission changes depending on the ratio of uninfected host individuals. When the ratio of uninfected hosts is low, infected individuals encounter each other more often than they encounter uninfected individuals, resulting in low horizontal transmission efficiency. At 37°C, horizontal transmission efficiency may decrease as the ratio of uninfected hosts decreases. During this period, the loss rate remains unaffected (i.e., does not decrease), allowing horizontal transmission and loss of AfuRV1 to be in dynamic equilibrium and stabilizing AfuRV1 prevalence before it reaches 100%. The opposite is also true when the ratio of uninfected hosts is high (Fig. S4). In the case of the AfuNV2 system, viral transmission and loss are rare. Therefore, the prevalence of the virus reached either 100% or 0%. In this study, it remains unclear whether the differences in prevalence outcomes between AfuRV1 and AfuNV2 are attributable to viral properties, host genotype, or experimental conditions.

Our results highlighted the analogy between persistent-type RNA viruses and plasmids. Previously, it was noted that plant endornavirus, a persistent-type RNA virus, has “plasmid-like properties,” primarily based on its persistence (31, 56). Our study adds that the prevalence of the virus fluctuates in response to environmental changes, suggesting population-level dynamics similar to those of plasmids. Plasmid prevalence also changes in response to environmental conditions such as temperature or antibiotic pressure (57–59). Furthermore, our results suggest that the primary factors driving the dynamics of persistent-type RNA viruses are akin to those of plasmids: horizontal transmission, loss, and impacts on host fitness (30). Although the mechanisms underlying each factor differ between plasmid and persistent-type RNA viruses, their population dynamics and driving forces are similar.

Based on phenotypic analysis, Roossinck et al. hypothesized that persistent RNA viruses contribute to the rapid adaptation of their hosts to environmental changes by generating epigenetic diversity in the host population (6, 9). Plasmids allow their host bacteria to adapt rapidly to environmental changes (58). Also, recent studies suggested that plasmids play a role in the adaptation of host bacteria to the local environment and niche differentiation by generating microdiversity of their host bacteria (60, 61). Therefore, the analogy between persistent-type RNA viruses and plasmids also implies the role of persistent RNA viruses as a contributor to host adaptation rather than simply molecular hitchhikers.

Virus-curing treatments have the potential risk to cause mutations in host genomes and alter host phenotypes. To tackle this, it is helpful to reintroduce the virus into the cured strain and use that for phenotypic comparisons instead of the original virus-infected strain (20, 62). As mentioned above, this study monitored transmission and loss, but the causes of each remain speculative. In some mycoviruses, viral accumulation levels vary depending on the growth temperature (63, 64). Therefore, temperature-dependent fluctuations in viral concentration and their molecular basis may be key factors underlying the observed differences in transmission efficiency and virus loss. However, it remains unclear whether these effects are directly caused by impairment of the viral replication function. Since this research was conducted under laboratory conditions, the relative strength of each factor cannot be estimated in real-world conditions. Additionally, it would be of interest to verify whether our framework also works in other organisms with different life cycles from fungi. Elucidating these points will help us understand why persistent RNA viruses exist in various eukaryotes.

## SUPPLEMENTAL MATERIAL

Supplemental material jvi.00582-25-s0001.docx Fig. S1 to S4; Tables S1 and S2.

ASM does not own the copyrights to Supplemental Material that may be linked to, or accessed through, an article. The authors have granted ASM a non-exclusive, world-wide license to publish the Supplemental Material files. Please contact the corresponding author directly for reuse.

## References

[1] Smith AE, Helenius A. 2004. How viruses enter animal cells. Science 304:237–242. doi:10.1126/science.1094823 15073366

[2] Bean AGD, Baker ML, Stewart CR, Cowled C, Deffrasnes C, Wang L-F, Lowenthal JW. 2013. Studying immunity to zoonotic diseases in the natural host - keeping it real. Nat Rev Immunol 13:851–861. doi:10.1038/nri3551 24157573 PMC7098194

[3] Mäntynen S, Salomaa MM, Poranen MM. 2023. Diversity and current classification of dsRNA bacteriophages. Viruses 15:2154. doi:10.3390/v15112154 38005832 PMC10674327

[4] García-Ordóñez L, Pagán I. 2024. Vertical and horizontal transmission of plant viruses: two extremes of a continuum? Npj Viruses 2:18. doi:10.1038/s44298-024-00030-8 40295758 PMC11721382

[5] Yu X, Zhu Y, Yin G, Wang Y, Shi X, Cheng G. 2024. Exploiting hosts and vectors: viral strategies for facilitating transmission. EMBO Rep 25:3187–3201. doi:10.1038/s44319-024-00214-6 39048750 PMC11315993

[6] Roossinck MJ. 2010. Lifestyles of plant viruses. Philos Trans R Soc Lond B Biol Sci 365:1899–1905. doi:10.1098/rstb.2010.0057 20478885 PMC2880111

[7] Roossinck MJ. 2014. Metagenomics of plant and fungal viruses reveals an abundance of persistent lifestyles. Front Microbiol 5:767. doi:10.3389/fmicb.2014.00767 25628611 PMC4290624

[8] Urayama S, Takaki Y, Chiba Y, Zhao Y, Kuroki M, Hagiwara D, Nunoura T. 2022. Eukaryotic microbial RNA viruses—acute or persistent? Insights into their function in the aquatic ecosystem. Microbes Environ 37:ME22034. doi:10.1264/jsme2.ME22034 35922920 PMC9763035

[9] Roossinck MJ. 2012. Persistent plant viruses: molecular hitchhikers or epigenetic elements. Viruses:177–186. doi:10.1007/978-94-007-4899-6

[10] Nuss DL. 2005. Hypovirulence: mycoviruses at the fungal-plant interface. Nat Rev Microbiol 3:632–642. doi:10.1038/nrmicro1206 16064055

[11] Ghabrial SA, Castón JR, Jiang D, Nibert ML, Suzuki N. 2015. 50-plus years of fungal viruses. Virology (Auckl) 479–480:356–368. doi:10.1016/j.virol.2015.02.034 25771805

[12] Myers JM, James TY. 2022. Mycoviruses. Curr Biol 32:R150–R155. doi:10.1016/j.cub.2022.01.049 35231405

[13] Urayama S, Zhao Y-J, Kuroki M, Chiba Y, Ninomiya A, Hagiwara D. 2024. Greetings from virologists to mycologists: a review outlining viruses that live in fungi. Mycoscience 65:1–11. doi:10.47371/mycosci.2023.11.004 39239117 PMC11371549

[14] Kotta-Loizou I, Coutts RHA. 2017. Mycoviruses in Aspergilli: a comprehensive review. Front Microbiol 8:1699. doi:10.3389/fmicb.2017.01699 28932216 PMC5592211

[15] Deng F, Xu R, Boland GJ. 2003. Hypovirulence-associated double-stranded RNA from Sclerotinia homoeocarpa is conspecific with Ophiostoma novo-ulmi mitovirus 3a-Ld. Phytopathology 93:1407–1414. doi:10.1094/PHYTO.2003.93.11.1407 18944069

[16] Khalifa ME, MacDiarmid RM. 2019. A novel totivirus naturally occurring in two different fungal genera. Front Microbiol 10:2318. doi:10.3389/fmicb.2019.02318 31681196 PMC6797558

[17] Deng Y, Zhou K, Wu M, Zhang J, Yang L, Chen W, Li G. 2022. Viral cross-class transmission results in disease of a phytopathogenic fungus. ISME J 16:2763–2774. doi:10.1038/s41396-022-01310-y 36045287 PMC9428384

[18] Niu Y, Yuan Y, Mao J, Yang Z, Cao Q, Zhang T, Wang S, Liu D. 2018. Characterization of two novel mycoviruses from Penicillium digitatum and the related fungicide resistance analysis. Sci Rep 8:5513. doi:10.1038/s41598-018-23807-3 29615698 PMC5882929

[19] Okada R, Ichinose S, Takeshita K, Urayama S, Fukuhara T, Komatsu K, Arie T, Ishihara A, Egusa M, Kodama M, Moriyama H. 2018. Molecular characterization of a novel mycovirus in Alternaria alternata manifesting two-sided effects: down-regulation of host growth and up-regulation of host plant pathogenicity. Virology (Auckl) 519:23–32. doi:10.1016/j.virol.2018.03.027 29631173

[20] Ninomiya A, Urayama S, Suo R, Itoi S, Fuji S-I, Moriyama H, Hagiwara D. 2020. Mycovirus-induced tenuazonic acid production in a rice blast fungus Magnaporthe oryzae Front Microbiol 11:1641. doi:10.3389/fmicb.2020.01641 32765467 PMC7379127

[21] Song H-Y, Kim N, Kim D-H, Kim J-M. 2020. The PoV mycovirus affects extracellular enzyme expression and fruiting body yield in the oyster mushroom, Pleurotus ostreatus. Sci Rep 10:1094. doi:10.1038/s41598-020-58016-4 31974404 PMC6978373

[22] Zhou L, Li X, Kotta-Loizou I, Dong K, Li S, Ni D, Hong N, Wang G, Xu W. 2021. A mycovirus modulates the endophytic and pathogenic traits of a plant associated fungus. ISME J 15:1893–1906. doi:10.1038/s41396-021-00892-3 33531623 PMC8245556

[23] Chu YM, Jeon JJ, Yea SJ, Kim YH, Yun SH, Lee YW, Kim KH. 2002. Double-stranded RNA mycovirus from Fusarium graminearum. Appl Environ Microbiol 68:2529–2534. doi:10.1128/AEM.68.5.2529-2534.2002 11976130 PMC127521

[24] Shah UA, Kotta-Loizou I, Fitt BD, Coutts RH. 2020. Mycovirus-induced hypervirulence of Leptosphaeria biglobosa enhances systemic acquired resistance to Leptosphaeria maculans in Brassica napus. Mol Plant-Microbe Interact 33:98–107. doi:10.1094/MPMI-09-19-0254-R 31652089

[25] Grassly NC, Fraser C. 2008. Mathematical models of infectious disease transmission. Nat Rev Microbiol 6:477–487. doi:10.1038/nrmicro1845 18533288 PMC7097581

[26] Reiner RC Jr, Perkins TA, Barker CM, Niu T, Chaves LF, Ellis AM, George DB, Le Menach A, Pulliam JRC, Bisanzio D, et al.. 2013. A systematic review of mathematical models of mosquito-borne pathogen transmission: 1970–2010. J R Soc Interface 10:20120921. doi:10.1098/rsif.2012.0921 23407571 PMC3627099

[27] Chowell G, Nishiura H. 2014. Transmission dynamics and control of Ebola virus disease (EVD): a review. BMC Med 12:196. doi:10.1186/s12916-014-0196-0 25300956 PMC4207625

[28] Hao X, Cheng S, Wu D, Wu T, Lin X, Wang C. 2020. Reconstruction of the full transmission dynamics of COVID-19 in Wuhan. Nature 584:420–424. doi:10.1038/s41586-020-2554-8 32674112

[29] Bao X, Roossinck MJ. 2013. A life history view of mutualistic viral symbioses: quantity or quality for cooperation? Curr Opin Microbiol 16:514–518. doi:10.1016/j.mib.2013.05.007 23796963

[30] Slater FR, Bailey MJ, Tett AJ, Turner SL. 2008. Progress towards understanding the fate of plasmids in bacterial communities. FEMS Microbiol Ecol 66:3–13. doi:10.1111/j.1574-6941.2008.00505.x 18507680

[31] Brown GG, Finnegan PM. 1989. RNA plasmids. Int Rev Cytol 117:1–56. doi:10.1016/s0074-7696(08)61333-9 2684889

[32] Hernández-Beltrán JCR, San Millán A, Fuentes-Hernández A, Peña-Miller R. 2021. Mathematical models of plasmid population dynamics. Front Microbiol 12:606396. doi:10.3389/fmicb.2021.606396 34803935 PMC8600371

[33] Chiba Y, Oiki S, Yaguchi T, Urayama S, Hagiwara D. 2021. Discovery of divided RdRp sequences and a hitherto unknown genomic complexity in fungal viruses. Virus Evol 7:veaa101. doi:10.1093/ve/veaa101 33505709 PMC7816673

[34] Yoshioka M, Fukudome A, Chiba Y, Hagiwara D, Urayama S. 2025. Characterization of a multi-segmented rod-shaped mycovirus within the order Martellivirales largely accommodating plant viruses. Virus Res 357:199591. doi:10.1016/j.virusres.2025.199591 40451551 PMC12169780

[35] Ikeda A, Chiba Y, Kuroki M, Urayama S, Hagiwara D. 2022. Efficient elimination of RNA mycoviruses in Aspergillus species using RdRp-inhibitors ribavirin and 2’-C-methylribonucleoside derivatives. Front Microbiol 13:1024933. doi:10.3389/fmicb.2022.1024933 36274709 PMC9583132

[36] Hagiwara D, Takahashi-Nakaguchi A, Toyotome T, Yoshimi A, Abe K, Kamei K, Gonoi T, Kawamoto S. 2013. NikA/TcsC histidine kinase is involved in conidiation, hyphal morphology, and responses to osmotic stress and antifungal chemicals in Aspergillus fumigatus. PLoS One 8:e80881. doi:10.1371/journal.pone.0080881 24312504 PMC3846623

[37] Barratt RW, Johnson GB, Ogata WN. 1965. Wild-type and mutant stocks of Aspergillus nidulans. Genetics 52:233–246. doi:10.1093/genetics/52.1.233 5857598 PMC1210840

[38] Urayama S, Katoh Y, Fukuhara T, Arie T, Moriyama H, Teraoka T. 2015. Rapid detection of Magnaporthe oryzae chrysovirus 1-A from fungal colonies on agar plates and lesions of rice blast. J Gen Plant Pathol 81:97–102. doi:10.1007/s10327-014-0567-6

[39] Odds FC, Brown AJP, Gow NAR. 2003. Antifungal agents: mechanisms of action. Trends Microbiol 11:272–279. doi:10.1016/s0966-842x(03)00117-3 12823944

[40] Hervé M. 2018. RVAideMemoire: testing and plotting procedures for biostatistics. R package version 09-69.

[41] Tekaia F, Latgé J-P. 2005. Aspergillus fumigatus: saprophyte or pathogen? Curr Opin Microbiol 8:385–392. doi:10.1016/j.mib.2005.06.017 16019255

[42] Márquez LM, Roossinck MJ. 2012. Do persistent RNA viruses fit the trade-off hypothesis of virulence evolution? Curr Opin Virol 2:556–560. doi:10.1016/j.coviro.2012.06.010 22819020

[43] Applen Clancey S, Ruchti F, LeibundGut-Landmann S, Heitman J, Ianiri G. 2020. A novel mycovirus evokes transcriptional rewiring in the fungus Malassezia and stimulates beta interferon production in macrophages. MBio 11:mBio doi:10.1128/mBio.01534-20 PMC7468202 32873760

[44] Roininen E, Vainio EJ, Sutela S, Poimala A, Kashif M, Piri T, Hantula J. 2024. Virus transmission frequencies in the pine root rot pathogen Heterobasidion annosum. Virus Res 350:199467. doi:10.1016/j.virusres.2024.199467 39299454 PMC11736397

[45] Dálya LB, Černý M, de la Peña M, Poimala A, Vainio EJ, Hantula J, Botella L. 2024. Diversity and impact of single-stranded RNA viruses in Czech Heterobasidion populations. mSystems 9:e0050624. doi:10.1128/msystems.00506-24 39287383 PMC11494978

[46] Fine PE. 1975. Vectors and vertical transmission: an epidemiologic perspective. Ann N Y Acad Sci 266:173–194. doi:10.1111/j.1749-6632.1975.tb35099.x 829470

[47] Ewald PW. 1983. Host-parasite relations, vectors, and the evolution of disease severity. Annu Rev Ecol Syst 14:465–485. doi:10.1146/annurev.es.14.110183.002341

[48] Ewald PW. 1987. Transmission modes and evolution of the parasitism-mutualism continuum. Ann N Y Acad Sci 503:295–306. doi:10.1111/j.1749-6632.1987.tb40616.x 3304078

[49] Schmitt MJ, Breinig F. 2006. Yeast viral killer toxins: lethality and self-protection. Nat Rev Microbiol 4:212–221. doi:10.1038/nrmicro1347 16489348

[50] Hyder R, Pennanen T, Hamberg L, Vainio EJ, Piri T, Hantula J. 2013. Two viruses of Heterobasidion confer beneficial, cryptic or detrimental effects to their hosts in different situations. Fungal Ecol 6:387–396. doi:10.1016/j.funeco.2013.05.005

[51] Filippou C, Diss RM, Daudu JO, Coutts RHA, Kotta-Loizou I. 2021. The polymycovirus-mediated growth enhancement of the entomopathogenic fungus Beauveria bassiana is dependent on carbon and nitrogen metabolism. Front Microbiol 12:606366. doi:10.3389/fmicb.2021.606366 33603722 PMC7884332

[52] Lipsitch M, Nowak MA, Ebert D, May RM. 1995. The population dynamics of vertically and horizontally transmitted parasites. Proc R Soc Lond B 260:321–327. doi:10.1098/rspb.1995.0099 7630898

[53] Lipsitch M, Siller S, Nowak MA. 1996. The evolution of virulence in pathogens with vertical and horizontal transmission. Evolution 50:1729–1741. doi:10.1111/j.1558-5646.1996.tb03560.x 28565576

[54] Bryner SF, Rigling D. 2012. Virulence not only costs but also benefits the transmission of a fungal virus. Evolution 66:2540–2550. doi:10.1111/j.1558-5646.2012.01637.x 22834751

[55] Hai D, Li J, Jiang D, Cheng J, Fu Y, Xiao X, Yin H, Lin Y, Chen T, Li B, Yu X, Cai Q, Chen W, Kotta-Loizou I, Xie J. 2024. Plants interfere with non-self recognition of a phytopathogenic fungus via proline accumulation to facilitate mycovirus transmission. Nat Commun 15:4748. doi:10.1038/s41467-024-49110-6 38834585 PMC11150657

[56] Fukuhara T, Koga R, Aoki N, Yuki C, Yamamoto N, Oyama N, Udagawa T, Horiuchi H, Miyazaki S, Higashi Y, Takeshita M, Ikeda K, Arakawa M, Matsumoto N, Moriyama H. 2006. The wide distribution of endornaviruses, large double-stranded RNA replicons with plasmid-like properties. Arch Virol 151:995–1002. doi:10.1007/s00705-005-0688-5 16341944

[57] Terawaki Y, Takayasu H, Akiba T. 1967. Thermosensitive replication of a kanamycin resistance factor. J Bacteriol 94:687–690. doi:10.1128/jb.94.3.687-690.1967 4166554 PMC251939

[58] Carattoli A. 2013. Plasmids and the spread of resistance. Int J Med Microbiol 303:298–304. doi:10.1016/j.ijmm.2013.02.001 23499304

[59] Wein T, Wang Y, Hülter NF, Hammerschmidt K, Dagan T. 2020. Antibiotics interfere with the evolution of plasmid stability. Curr Biol 30:3841–3847. doi:10.1016/j.cub.2020.07.019 32795438

[60] Finks SS, Martiny JBH. 2023. Plasmid-encoded traits vary across environments. mBio 14:e0319122. doi:10.1128/mbio.03191-22 36629415 PMC9973032

[61] Finks SS, Moudgalya P, Weihe C, Martiny JBH. 2024. The contribution of plasmids to trait diversity in a soil bacterium. ISME Commun 4:ycae025. doi:10.1093/ismeco/ycae025 38584646 PMC10999282

[62] Liu H, Wang H, Liao XL, Gao B, Lu X, Sun D, Gong W, Zhong J, Zhu H, Pan X, Guo L, Deng XW, Zhou Q. 2022. Mycoviral gene integration converts a plant pathogenic fungus into a biocontrol agent. Proc Natl Acad Sci USA 119. doi:10.1073/pnas.2214096119 PMC9897477 36469771

[63] Amin H, Zamora-Ballesteros C, Diez-Casero JJ. 2025. Effects of thermal and antibiotic treatments on the viral accumulation of FcMV1 in Fusarium circinatum isolates. J Fungi (Basel) 11:267. doi:10.3390/jof11040267 40278088 PMC12027980

[64] Romon-Ochoa P, Smith O, Lewis A, Kupper Q, Shamsi W, Rigling D, Pérez-Sierra A, Ward L. 2023. Temperature effects on the Cryphonectria hypovirus 1 accumulation and recovery within its fungal host, the chestnut blight pathogen Cryphonectria parasitica Viruses 15:1260. doi:10.3390/v15061260 37376560 PMC10304158

