# Supplementary material for: Plasmid-like dynamics of persistent RNA viruses in the host fungal population
Source: J Virol. 2025 Jul 31;99(8):e00582-25. doi: 10.1128/jvi.00582-25 (PMC12363214; doi:10.1128/jvi.00582-25)
Supplement: Supplemental material — Fig. S1 to S4; Tables S1 and S2. [file jvi.00582-25-s0001.docx]

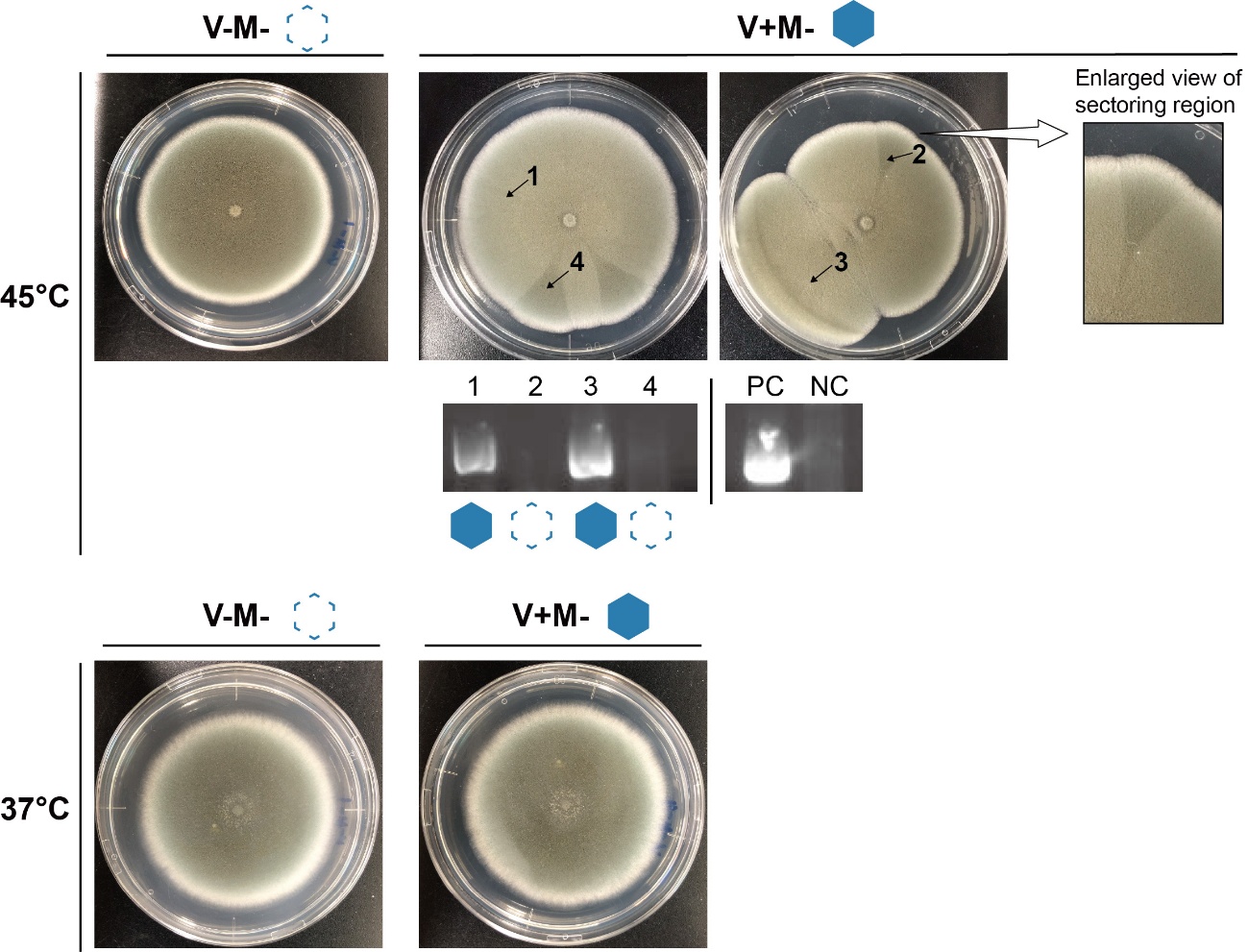


Fig. S1: Colony morphology of AfuRV1-infected strain under two different temperatures and detection of AfuRV1 from a colony cultured at 45°C. The numbers written in the pictures of colonies correspond to the number displayed above the agarose-gel electrophoresis image. Total nucleic acids extracted from AfuRV1-infected and -free strains were used as the positive control (PC) and negative control (NC), respectively.


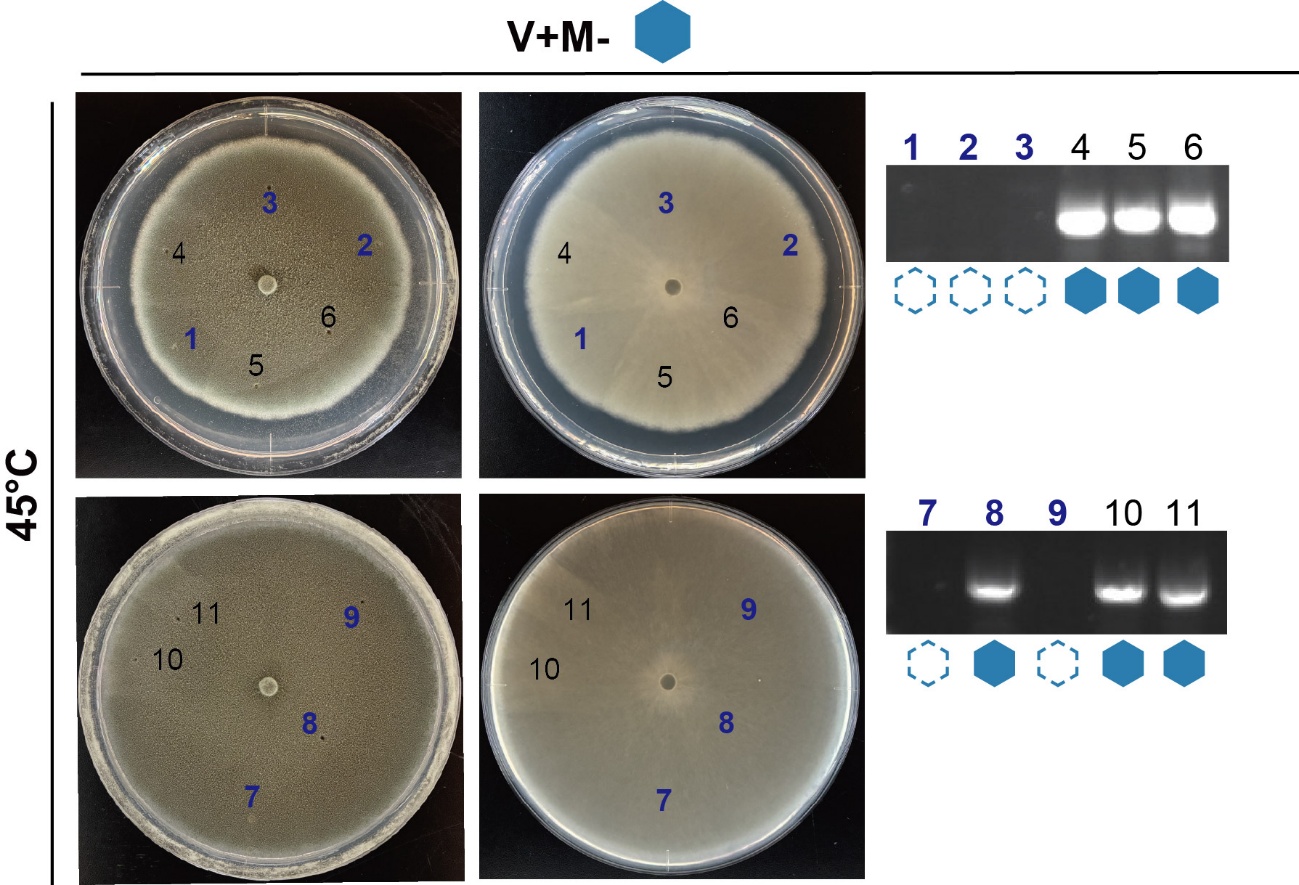


Fig. S2: Colony morphology of AfuRV1-infected strain obtained through single-spore isolation and detection of AfuRV1 from the colony. The numbers written in the pictures of colonies correspond to the number displayed above the agarose-gel electrophoresis image. The Bold Blue font indicates the sectoring region. For single-spore isolation, we spread 200 μL of the spore suspension (containing approximately 20 spores) on PDA. After overnight incubation at 45°C, the single colonies that emerged were transferred one by one to new PDA plates and incubated at 45°C. Virus detection was conducted as described in the Materials and Methods.


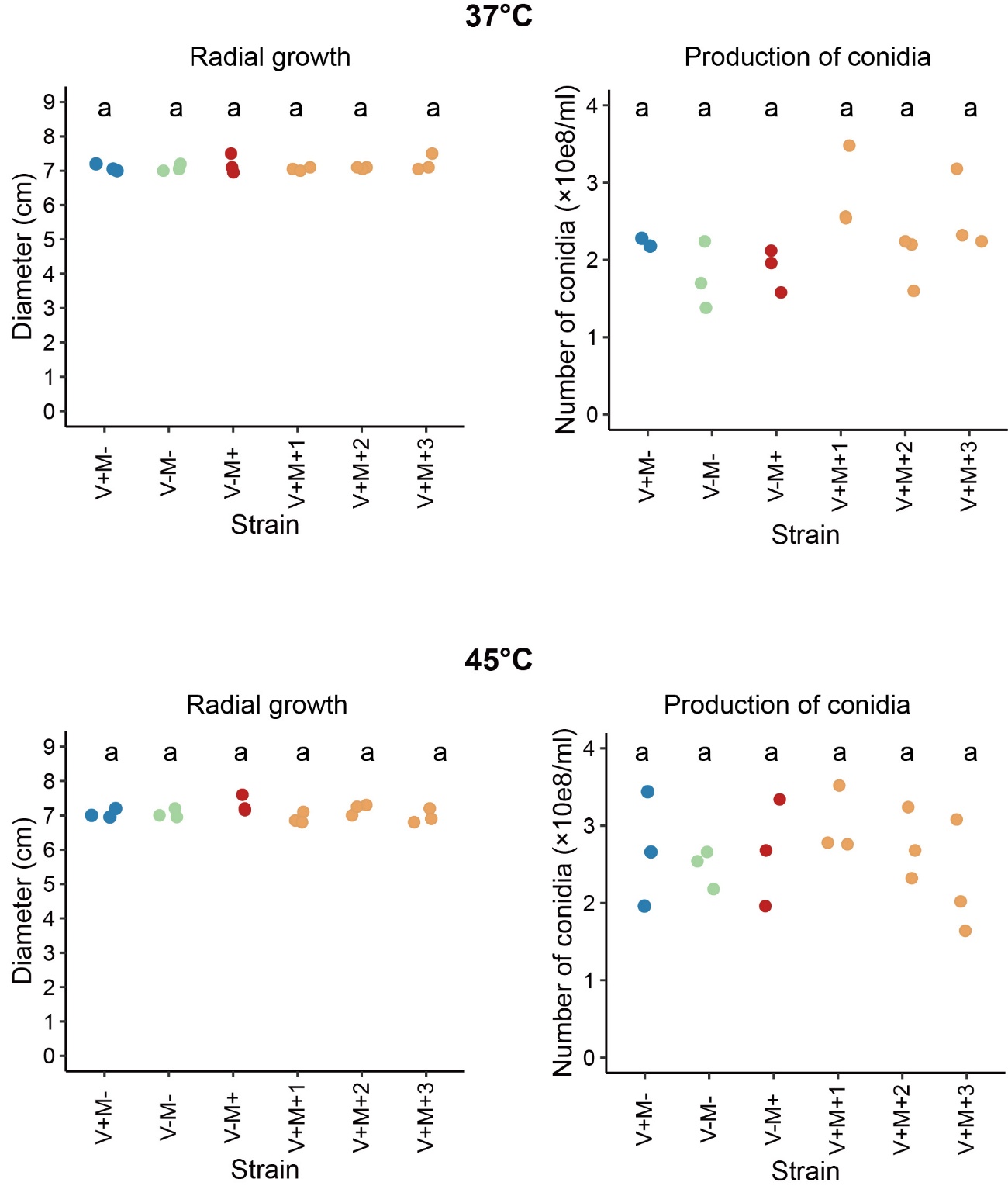


Fig. S3: Growth assay of four AfuRV1-related strains at 37°C or 45°C. Three independent cultures were examined. Dots indicate each biological replicate. The same letters above the dots indicate no significant pairwise differences (Tukey’s HSD, p > 0.05).


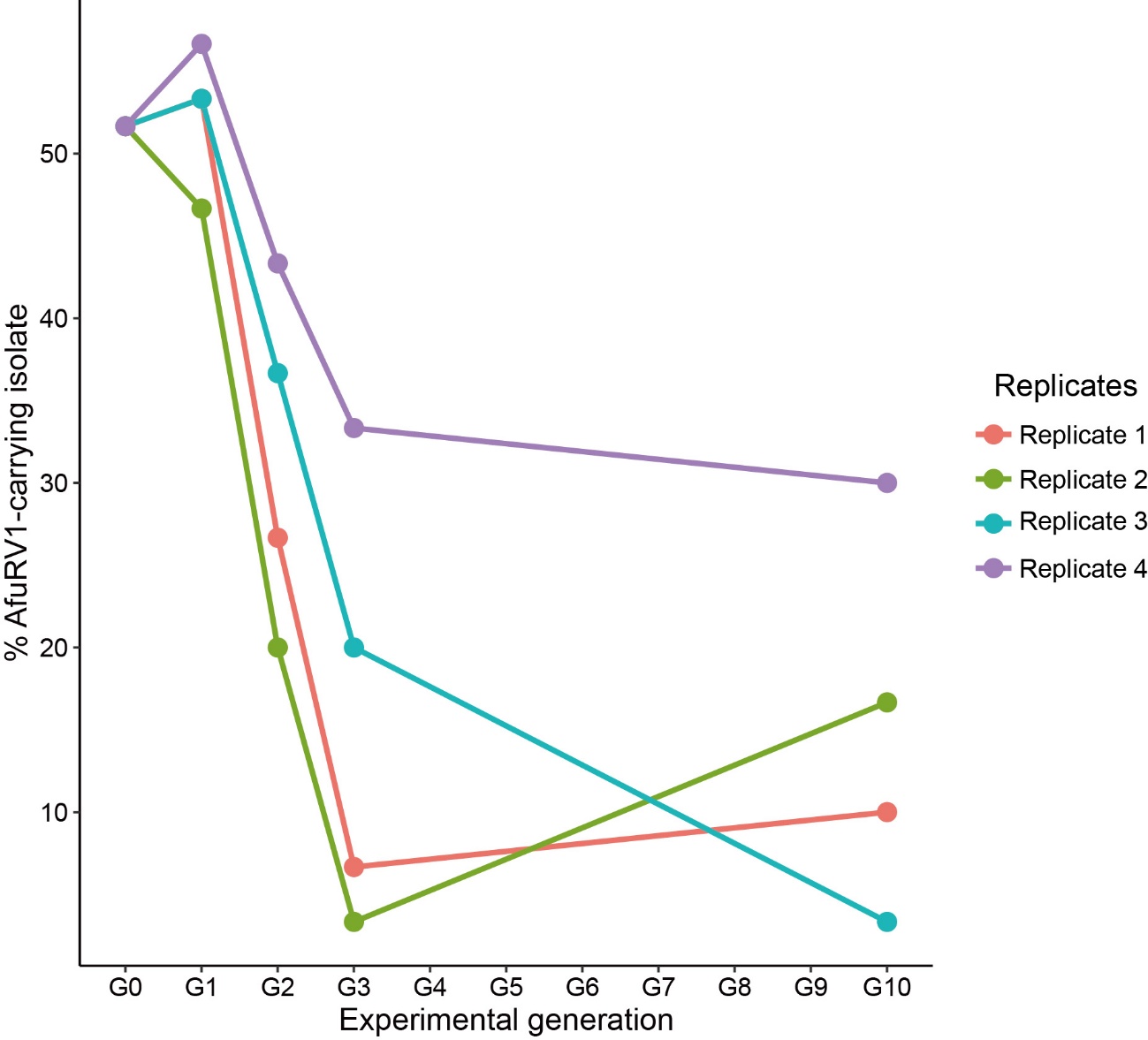


Fig. S4: Dynamics of AfuRV1 prevalence in long-term cultivation at 45°C. Culture of the populations shown in Fig. 1B was continued by 10 experimental generations at 45°C. Lines connect each of the four replicate populations across time. AfuRV1 prevalence was observed in time points with dots.

Table S1: Vertical transmission (VT) rate of AfuRV1 and AfuNV2

| Tested Virus | Condition | Replicates* | Virus prevalence in spores (VT rate) |
| --- | --- | --- | --- |
| AfuRV1 | 37℃ | Replicate 1 | 29/30 (96.7%) |
|  |  | Replicate 2 | 28/30 (93.3%) |
|  |  | Replicate 3 | 29/30 (96.7%) |
|  |  | Total | 86/90 (95.6%) |
|  | 45℃ | Replicate 1 | 28/30 (93.3%) |
|  |  | Replicate 2 | 26/30 (80%) |
|  |  | Replicate 3 | 30/30 (100%) |
|  |  | Total | 82/90 (91.1%) |
| AfuNV2 | 37℃ | Replicate 1 | 29/30 (96.7%) |
|  |  | Replicate 2 | 30/30 (100%) |
|  |  | Replicate 3 | 30/30 (100%) |
|  |  | Total | 89/90 (98.9%) |

* We used multiple distinct virus-infected colonies obtained through single-spore isolation as biological replicates in the experiments. For AfuRV1 at 45°C, we collected mycelial plugs from non-sectoring regions in the colonies prepared in the experiment shown in Fig. S2. For AfuRV1 at 37°C and AfuNV2, we prepared colonies and collected plugs in the same manner as for AfuRV1 at 45°C, except for culture temperature. Then, we harvested spores from the plugs with 0.05% Tween 20 for observation of virus prevalence. Virus prevalence was majored as described in the Materials and Methods.

Table S2: List of primers used in this study

| Targeted gene | Primer name | Sequence (5'-3') | Product size (bp) |
| --- | --- | --- | --- |
| AfuRV1 RdRp | afmix2_luck_3F | TTGCCGCTTCGGGCTGCTCG | 645 |
|  | afmix2_luck_3R | CGCGTAGTACCACATCCTGC |  |
| AfuNV2 RdRp | AfuNV2-1,2_1F | CTCGAGGTTATCCCGAGTAG | 499 |
|  | AfuNV2-1,2_1R | CAGACCTAAGTCCGTGTCGT |  |
| Hyg^R^ gene | hph_qpcr_P1F | ACAATGGCCGCATAACAGCG | 225 |
|  | hph_qpcr_P1R | TGCCGTCAACCAAGCTCTGA |  |
